# Cheminformatics Identification and Validation of Dipeptidyl Peptidase-IV Modulators from Shikimate Pathway-Derived Phenolic Acids towards Interventive Type-2 Diabetes Therapy

**DOI:** 10.3390/metabo12100937

**Published:** 2022-10-02

**Authors:** Fatai Oladunni Balogun, Kaylene Naidoo, Jamiu Olaseni Aribisala, Charlene Pillay, Saheed Sabiu

**Affiliations:** Department of Biotechnology and Food Science, Faculty of Applied Science, Durban University of Technology, Durban 4001, South Africa

**Keywords:** chlorogenic acid, diprotin A, dipeptidyl peptidase IV, molecular dynamics simulations, phenolic acids, type-2 diabetes mellitus

## Abstract

Recently, dipeptidyl peptidase-IV (DPP-IV) has become an effective target in the management of type-2 diabetes mellitus (T2D). The study aimed to determine the efficacy of shikimate pathway-derived phenolic acids as potential DPP-IV modulators in the management of T2D. The study explored in silico (molecular docking and dynamics simulations) and in vitro (DPP-IV inhibitory and kinetics assays) approaches. Molecular docking findings revealed chlorogenic acid (CA) among the examined 22 phenolic acids with the highest negative binding energy (−9.0 kcal/mol) showing a greater affinity for DPP-IV relative to the standard, Diprotin A (−6.6 kcal/mol). The result was corroborated by MD simulation where it had a higher affinity (−27.58 kcal/mol) forming a more stable complex with DPP-IV than Diprotin A (−12.68 kcal/mol). These findings were consistent with in vitro investigation where it uncompetitively inhibited DPP-IV having a lower IC_50_ (0.3 mg/mL) compared to Diprotin A (0.5 mg/mL). While CA showed promising results as a DPP-IV inhibitor, the findings from the study highlighted the significance of medicinal plants particularly shikimate-derived phenolic compounds as potential alternatives to synthetic drugs in the effective management of T2DM. Further studies, such as derivatisation for enhanced activity and in vivo evaluation are suggested to realize its full potential in T2D therapy.

## 1. Introduction

Diabetes mellitus is a popular metabolic derangement whose annual impact continues to grow globally; it is anticipated that by 2030, over 764 million people will be suffering from the disease (if no suitable solution is found) [1] as compared to 463 million in 2019 [2]. Type-2 diabetes mellitus (T2D) is characterised by hyperglycaemia resulting from insulin resistance, insensitivity, or both [2,3]. It is noteworthy that prolonged hyperglycaemic conditions in a diabetic state could warrant (excessive) glucose binding covalently to plasma protein (glycation) causing a breakdown of receptor function or alteration of some enzymatic activities. Thus, this leads to the emergence of diabetic complications, such as impairment of vision (retinopathy), kidney dysfunction (nephropathy), and nerve damage (neuropathy), among others. Hence, the ultimate goal for any T2D therapy is to lower the systemic glucose level, and thus prevent the emergence of possible associated diseases, such as hypertension, stroke, etc. [4]. Management options including the use of oral hypoglycaemic agents (OHAs), such as sulphonylureas, biguanides, etc., and insulin have been adopted [3,4,5]. Sadly, these agents only bring-about normoglycaemia in half of the diabetes sufferers [6]; additionally, they are also characterised by side effects (e.g., obesity, hypoglycaemia, hormonal imbalance, etc.), thus, the clamor for potential alternatives in natural products which are cost-effective and with relatively minimal side effects [7,8].

One of the viable approaches to controlling T2D is via glucagon-like peptide 1 (GLP-1) concerned with the potential of reducing fasting and postprandial glucose in the blood for T2D [3,9,10]. Though, GLP-1 is continuously degraded in the systemic circulation by dipeptidyl peptidase-IV (DPP-IV), a serine aminopeptidase enzyme, inhibitors of DPP-IV would come in handy in preventing this breakdown and thus ensures continuous survival of GLP-1 [3]. Dipeptidyl peptidase-IV (DPP-IV) inhibitors and glucagon-like peptide 1 (GLP-1) are a newer class of antidiabetics [4,6,11]. Dipeptidyl peptidase-IV, widely distributed in tissues and sites (liver, kidney, bone marrow, blood vessels, endo- and/or epithelial cells, etc.) is involved in the inactivation of GLP-1 and glucose-dependent insulinotropic polypeptide (GIP) concerned in the heightened biosynthesis of insulin, the proliferation of β-cells, and the reduction of β-cell apoptosis resulting from postprandial glucose episodes [4]. Hence, inhibiting the action of DPP-IV has been regarded as an important mechanism for systemic glucose control [11,12]. Phenolic acid-derived natural products have been studied and established to elicit various pharmacological effects, such as antioxidative, anticancer, antidiabetic, etc. [3,13,14,15]. Interestingly, reports of phenolic compounds, such as cyanidin, kaempferol, quercitin, hesperetin, genistein, naringenin, chrysin, methyl p-coumarate as DPP-IV inhibitors from natural products, such as soybeans, grape, *Melicope latifolia*, and citrus have been reported [13,14,15,16]. There are numerous submitted in vitro studies that assessed the effects of phytocompounds on glucose reduction via DPP-IV inhibition [13,14,15,16], in fact, an in vivo study has also established a reduction in the level of glucose in streptozotocin-induced diabetic rats by the phenolic compound, isoquercitrin isolated from *Apocynum cannabinum* leaves and *Gossypium herbaceum* flower indicating its effectiveness as a DPP-IV inhibitor [17]. However, since there is a dearth of information on the shikimate pathway-derived phenolic acids against DPP-IV, it is envisaged that studying the effect of shikimate pathway-derived phenolic acids as DPP-IV inhibitors could be promising in the development of drug candidates in T2D therapy.

Computational methods have been extensively used to study the interactions between a protein and complementary ligands [18]. The methods can be the prelude step during drug discovery to screen a library of compounds in search of promising bioactive principles with the best therapeutic effect. The methods allow for the elimination of undesirable compounds prior to in vitro and in vivo analyses [18]. Hence, employing computational methods in this study should provide insight into which of the shikimate pathway-derived phenolic acids would afford better interactions with DPP-IV to elicit an effective therapeutic response towards drug discovery prior to in vitro analyses. Based on this background, this study was conceptualised to assess the potential of phenolic acids derived from the shikimate pathway that could be further developed as probable drug candidates against DPP-IV in the management of T2D and to reveal the possible mechanism of inhibition depicted by the best compound through in silico and in vitro methods.

## 2. Materials and Methods

### 2.1. Chemicals and Reagents

Chlorogenic acid (≥95%), human DPP-IV, Gly-Pro-4-methoxy-β-naphthylamide (≥98%), and Diprotin A (≥97%) were obtained from Sigma-Aldrich (St. Louis, MO, USA). Unnamed chemicals used for reagent preparation were of analytical grade.

### 2.2. Methodology

#### 2.2.1. In Silico Study

##### Molecular Docking

The 22 phenolic acids (chlorogenic acid, ellagic acid, gallic acid, caffeic acid, o-coumaric acid, olivetolic acid, umbellic acid, isoferulic acid, m-coumaric acid, p-coumaric acid, protocatechuic acid, orsellinic acid, ferulic acid, sinapic acid, hypogallic acid, vanillic acid, β-resorcinolic acid, salicyclic acid, syringic acid, veratric acid, gentisic acid, benzoic acid) mined from the shikimate pathway were subjected to molecular docking (MD) adopting the method of Ibrahim et al. [19]. Briefly, the X-ray crystal structure of DPP-IV (ID: 1WCY) was downloaded in PDB format from the Protein Data Bank (https://www.rcsb.org, accessed on 2 August 2021) (Research Collaboratory for Structural Bioinformatics, University of California, San Diego, CA, USA) and optimised using Chimera v1.15 (Resource for Biocomputing, Visualization and Informatics, University of California, San Francisco, CA, USA). The optimisation involves the removal of the co-crystallised ligand and non-standard residues and saving in PDB format for further analyses. PubChem (National Centre for Biotechnology Information, Bethesda, MD, USA) downloaded (https://pubchem.ncbi.nlm.nih.gov, accessed on 2 August 2021) structures of the 22 phenolic acids and Diprotin A three-dimensional (3D), were prepared in Chimera v1.15 software by adding non-polar H atoms and Gasteiger charges. The prepared compounds (optimised) were saved in mol2 format upon being opened in Chimera v1.15 [20]. The PDB version of the DPP-IV and the mol2 version of the phenolic acids and standard were thereafter, subjected to the Autodock Tool (The Scripps Research Institute, San Diego, CA, USA) [21] with all parameters in default settings. The grid sizes were set, and the grid centers were designated at specific dimensions (Centre (Å): 42.4291; 64.2981; 29.9357, Size (Å): 44.834; 34.693; 74.9341, corresponding to x, y, z coordinates, respectively). Based on the docking scores, complexes with the best pose for each phenolic acid were chosen and ranked. The most promising phenolic acid complex was viewed in Discovery Studio v21 to establish the nature of interactions with the amino acid residues of the enzyme at the active site.

**Figure 1:**
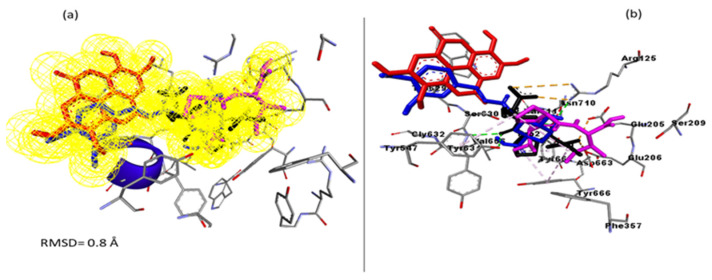
Validation of docking technique and parameters via the superimposition approach against the co-crystal structure of Dipeptidyl peptidase-IV (1WCY). (**a**) The superimposition showed that the docked Diprotin A (purple colour), chlorogenic acid (blue colour), and ellagic acid (red colour) achieved the same orientation with the native inhibitor (black) of 1WCY with a low RMSD value of 0.8 Å. (**b**) showed the investigated compounds and native inhibitors at the binding pocket of DPP-IV displaying active site amino acids.

However, as molecular docking methods often produce pseudo-positive binding conformations as the most energy-minimised pose, validation of the docking study was conducted by measuring the Root Mean Square Deviation (RMSD) of the docked ligands from the reference position (native inhibitor) in the experimental co-crystal structure of DPP-1V (1WCY) after optimal superimposition [22]. A low RMSD value of 0.8 Å between the docked ligands from the native inhibitor in the crystal structure of DPP-1V suggests the same binding orientation, which lent credence to the docking technique employed (Figure 1).

##### Molecular Dynamics Simulations

The molecular dynamics simulation (MDS) carried out in 100 ns was performed on the most promising phenolic acid complex as previously reported by Sabiu et al. [23] using the GPU version within the AMBER package. The AMBER LEaP module was neutralised with the inclusion of H atoms and Na^+^ and Cl^−^ counter ions. The system in each case was dipped implicitly within an orthorhombic box of TIP3P water molecules such that all atoms were within 8Å of any box edge. For each simulation, the SHAKE algorithm was used to constrict the hydrogen (H) atoms. The simulations align with the isobaric-isothermal ensemble (NPT), having randomised seeding, 2 ps pressure-coupling constant, 27 Berendsen barostat maintains 1 bar constant pressure, 300 K temperature and Langevin thermostat with a collision frequency of 1.0 ps [24]. Using PTRAJ, the systems were subsequently saved, and each trajectory was analyzed every 1 ps, and the RMSD, Radius of Gyration (RoG), Root Mean Square Fluctuation, Solvent Accessible Surface Area (SASA), and the H-bond flexibility were analysed with AMBER 18 suite of CPPTRAJ module. The Molecular Mechanics/GB Surface Area method (MM/GBSA) was used for the analysis of the negative free binding energy (∆G) over 100,000 snapshots extracted from the 100 ns trajectory [25].

##### Pharmacokinetics Assessment

The pharmacokinetic properties comprising the absorption, distribution, metabolism, excretion and toxicity (ADMET) and drug-likeness features of the compound with the most promising complex were predicted with the SWISS ADME (Swiss Institute of Bioinformatics, Lausanne, Switzerland) server (http://swissadme.ch/index.php, accessed on 5 September 2021) while the prediction of probable toxicity was achieved using ProTox (https://tox-new.charite.de/protox_II/, accessed on 6 September 2021) (Structural Bioinformatics Group, CUMIP, Berlin, Germany) [26].

#### 2.2.2. In Vitro Evaluation

##### DPP-IV Inhibitory Assay

The compound (chlorogenic acid) with the best binding affinity towards DPP-IV was evaluated in vitro and was carried out as reported by Oliveira et al. [27] with modifications. The spectrophotometric assay was based on Gly-Pro-4-methoxy-β-naphthylamide, a chromogenic synthetic DPP-IV substrate, cleavage to β-naphthylamine. In brief, 10 mg chlorogenic acid was suspended in 10 mL of Tris-HCl buffer (50 mM, pH 7.5) in a 15 mL microcentrifuge tube to yield a 1 mg/mL stock solution from where varying concentrations (0.50, 0.40, 0.30, 0.20, 0.10, and 0.05 mg/mL) were prepared. Thereafter, 50 µL of Tris-HCl buffer, 20 µL of DPP-IV (at a final concentration of 17.34 µU/µL in Tris-HCl buffer) and 50 µL of various concentrations of DPP-IV and Diprotin A were released into a 96-well microtiter plate wells and incubated at 37 °C for 10 min. Subsequently, 50 µL of the substrate (0.2 mM in Tris-HCl buffer) was added and further incubated for 30 min at 37 °C. The absorbance readings were taken using a microtiter plate reader (OPTIZEN POP, Apex Scientific, Durban, South Africa) at 405 nm. The experiment was conducted in triplicate and the negative control had no chlorogenic acid. The inhibitory percentage was obtained following the expression [Abs_0_ − bs1/Abs_0_ × 100 (where Abs_0_ is the absorbance of the blank and Abs_1_ is the absorbance of either chlorogenic acid or Diprotin A) and thereafter converted to half-maximal inhibitory concentration (IC_50_) using a linear calibration curve.

##### Enzyme Kinetics Evaluation

The mode of inhibition of DPP-IV by chlorogenic acid was determined as previously described by Jung et al. [28]. Briefly, 20 µL chlorogenic acid (IC_50_: 0.3 mg/mL) and DPP-IV were dispensed into five wells of a 96-well plate for a set of experiments. In another set of the experiment (control), a mixture of the buffer (without chlorogenic acid) and DPP-IV with the same volume as the earlier set was prepared. Following this in ascending order, various concentrations (0.1–0.5 mM) of Gly-Pro-4-methoxy-β-naphthylamide were added to the two sets of experiments and subsequently incubated for 20 min at 37 °C. While the experiment was carried out in triplicate, the absorbance of the reacting mixtures was measured at 405 nm using a spectrophotometer (OPTIZEN POP, Apex Scientific, Durban, South Africa) and the calibration curve of Gly-Pro-4-methoxy-β-naphthylamide was used to calculate the amount of produced product (β-naphthylamine) over time and subsequently converted to reaction velocities. A double reciprocal plot of reaction rates (1/V) and substrate concentrations (1/[S]) [29] was carried out for the generation of V_max_, K_m_ and K_cat_ parameters of chlorogenic acid.

### 2.3. Statistical Analysis

Data from the in vitro analysis were analysed using GraphPad Prism 9.2.0 (GraphPad, La Jolla, CA, USA) using a *t*-test (and nonparametric tests), supplemented with a Mann–Whitney test. Results are presented as mean ± standard deviation (SD) of triplicate determinations (n = 3) and mean with *p*-value < 0.05 were considered significant and statistically different. Raw data were analyzed with Origin software V18 (OriginLab Corporation, Northampton, MA, USA) for the in-silico evaluations [30].

## 3. Results and Discussion

### 3.1. Computational Analysis

Molecular docking examines the affinity and poses of a compound at an enzyme’s active site; compound(s) with the most negative binding score indicate better-posed compounds and a prospective modulator of the enzyme [23]. In this study, the results of the MD of the 22 phenolic compounds against DPP-IV revealed chlorogenic acid as the most promising compound judging by its docking score of −9.0 kcal/mol followed by ellagic acid (−7.8 kcal/mol) relative to the reference standard, Diprotin A, with a score of −6.6 kcal/mol (Table 1). It should be noted that the most negative docking scores of chlorogenic acid and ellagic acid, depicting their better pose in comparison to other compounds and standards, necessitated their further consideration in the study.

Interestingly, the observation with chlorogenic acid regarding its docking score in this study is noteworthy as it interacted more favorably with DPP-IV than other phenolic compounds, such as quercetin and coumarin, previously evaluated against the enzyme [3]. A further probe into the energy component profiles of the chlorogenic acid–DPP-IV and ellagic acid–DPP-IV complexes after the 100 ns MDS further supported the affinity of DPP-IV for chlorogenic acid where its overall binding energy was comparably higher (*p* > 0.05) (−25.74 kcal/mol) compared with ellagic acid (−24.54 kcal/mol) relative to Diprotin A–DPP-IV complex, which was the lowest (*p* < 0.05) (−12.44 kcal/mol) (Table 2).

**Figure 2:**
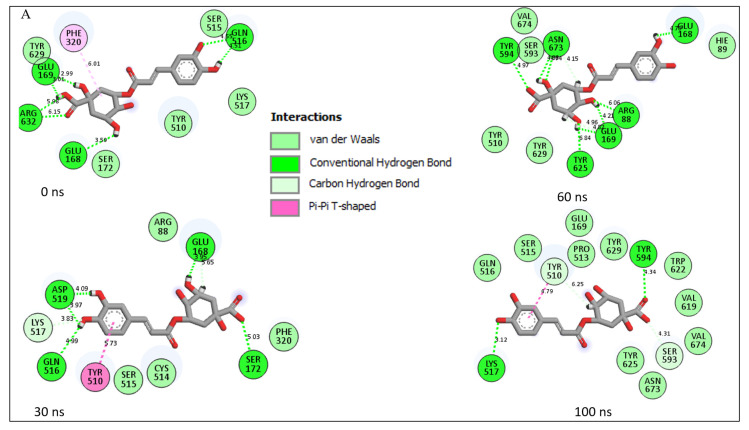

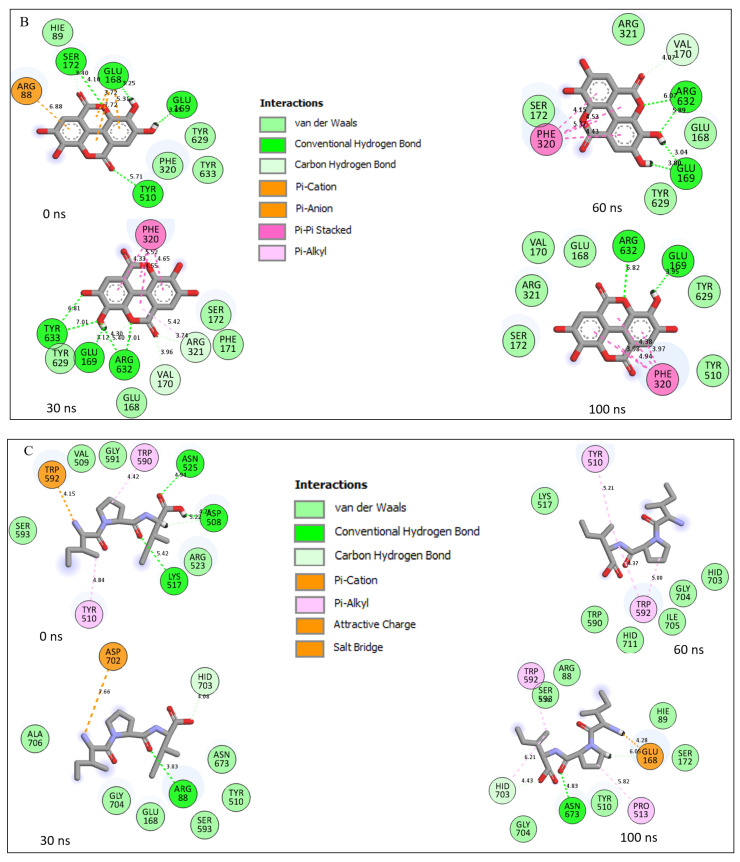
Interaction types and plots of (**A**) Chlorogenic acid–DPP-IV; (**B**) Ellagic acid–DPP-IV and (**C**) Diprotin A–DPP-IV at different time frame during a 100 ns simulation.

An understanding of the nature of interaction established between the amino acids at the active site of a protein with a ligand is crucial in the development of the ligand as a drug candidate [31]. In this study, chlorogenic acid, ellagic acid and Diprotin A interacted with Ser630 and His740 of the catalytic triad at the active site of DPP-IV (Figure 2) and this observation was in line with the work of Poonam et al. [14] where Ser630 was identified as one of the amino acid residues through which chrysin interacted with DPP-IV. Interaction with the catalytic triad is characteristic of potential DPP-IV inhibitors, preventing endogenous peptides, such as GLP-1 and GIP from interacting with the active site, thus, hindering their subsequent cleavage [32]. The findings from this study regarding the modulatory role of chlorogenic acid on the active site amino acid residues of DPP-IV could provide insights into the possible mechanisms through which chlorogenic acid increased GLP-1 levels, improved the glycaemic index [33], and improved glucose homeostasis and insulin resistance in vivo [34], as previously reported.

Furthermore, the type and number of interactions formed as a result of the binding between the ligand and the amino acid residues of the target protein are crucial in determining the extent of the resulting affinity [35]. In this study, 14 interactions comprising two conventional H-bond (Lys517 and Tyr594), 10 van der Waal forces (Tyr625, Asn673, Val674, Val619, Trp622, Tyr629, Pro513, Glu169, Ser515, Gln516) and two carbon-H bonds (Ser593, Tyr510) were formed with the complexation of chlorogenic acid and DPP-IV (Figure 2A), which is in sharp contrast to the nine interactions [two conventional H-bonds (Arg632 and Glu169), six van der Waal forces (Ser172, Arg321, Val170, Glu168, Tyr629 and Tyr510) and one π-π stacked (Phe320)] from the ellagic–DPP-IV complex (Figure 2B) and eleven interactions [one conventional H-bond (Asn673), six vans der Waal forces (Gly704, Tyr510, Ser172, Hie89, Ser598, Arg88), one carbon-H bond (Hid703), two π-alkyl (Pro513, Trp592) and one salt bridge (Glu168)] observed for the Diprotin A–DPP-IV complex (Figure 2C). The interactions depicted by the three systems are in conformity with the results of the thermodynamics profiles except between ellagic acid and Diprotin A, where the ellagic acid–DPP-IV complex with the most negative binding energy value (which might have been contributed by the two H-bonds) depicted fewer numbers of interactions in comparison with Diprotin A with an H-bond, since it has been reported that H-bonds contribute higher energies to the complex [36]. Additionally, the lowest number of interactions depicted by few numbers of conventional H and van der Waal bonds for the Diprotin A–DPP-IV complex might be suggested as the reason for its lower binding energy score compared to chlorogenic acid–DPP-IV with higher binding energy characterised by a greater number of convention H-bond and van der Waal forces. The higher number of interactions and H-bonds witnessed in this study by chlorogenic acid over Diprotin A was in line with similar work by Tuersuntuoheti et al. [37] where chlorogenic acid isolated from Qingke barley fresh noodles had 19 interactions (nine H-bonds and 10 hydrophobic interactions with amino acids residues of DPP-IV) compared with the used standard (sitagliptin) having 15 interactions (one H-bond and 14 hydrophobic interactions). During the 100 ns simulation period, the conserved residue Tyr510 in the interaction plots of Diprotin A and chlorogenic acid with DPP-IV in each of the time frames studied is relevant to the affinity and stability observed with the two compounds towards the target (Figure 2A,C). This crucial amino acid is missing in the 30 ns and 60 ns time frames in the interaction plot of ellagic acid with DPP-IV (Figure 2C) and may have contributed to the reduced stability observed with ellagic acid compared to the other compound studied with DPP-1V.

Binding energy reflects the totality of all the intermolecular forces or ligand and target interactions and the degree of binding occurring between ligand–protein. It has been reported to be enhanced by H-bonds [38], thus, further corroborating our submission in this study. Additionally, the π-alkyl bond was also among the interactions found in the Diprotin A–DPP-IV complex, possibly contributing to its reduced binding energy. Thus, this finding could be said to be consistent with a previous report [23] where the presence of π-cation interactions of the alpha-glucosidase-acarbose complex was attributed to its lesser binding activity.

**Figure 3:**
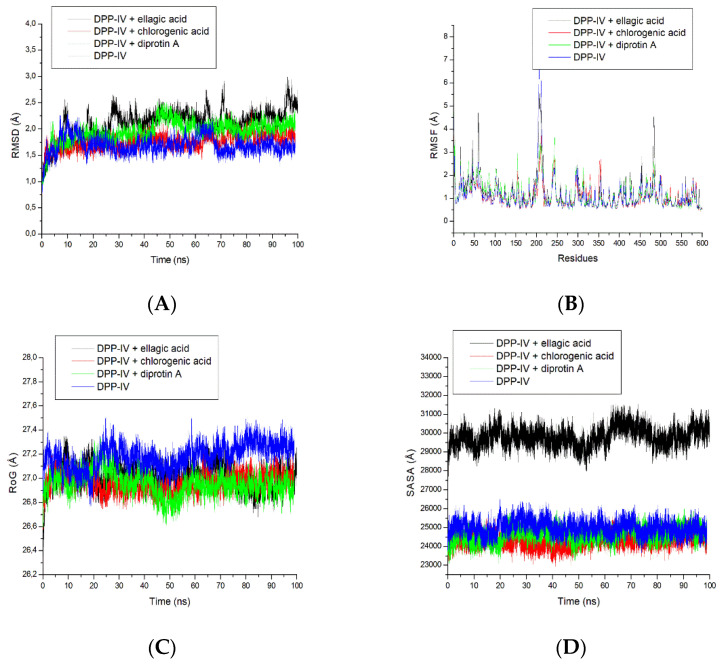
(**A**) Root Mean Square Deviation (RMSD), (**B**) Root Mean Square Fluctuations (RMSF), (**C**) Radius of Gyration (RoG), and (**D**) Solvent Accessible Surface Area (SASA) plots of comparison between Dipeptidyl peptidase-IV (DPP-IV) and chlorogenic acid, ellagic acid and Diprotin A determined over 100 ns molecular dynamics simulations.

Molecular dynamics simulation explores potential changes in the stability, structure, or conformation of a protein–ligand complex following ligand binding [39]. It is, therefore, germane to study parameters, such as RMSD, RMSF, RoG, SASA, and H-bond fluctuations defining whether the overall stability of the complex is maintained. The RMSD measures the changes that the enzyme–ligand structure undergoes over time, and it provides information on the overall stability of the complex [40]. The result of this investigation revealed an average RMSD of DPP-IV after 100 ns to be 1.69 Å while that of chlorogenic acid–DPP-IV, Diprotin A–DPP-IV and ellagic acid–DPP-IV complexes had average RMSD values of 1.75 Å, 1.95 Å and 2.10 Å, respectively (Supplemental Table S1). A lower average RMSD corresponds to a more stable complex over the simulation period [40]. In this study, the mean RMSD values of the three complexes are higher than that of the unbound DPP-IV; however, this might not be taken as an indication of the instability of the complexes during the simulation period. In fact, Rosenberg [41] reported that an RMSD exceeding 3.5 Å may only indicate an unstable complex and an unsuitable inhibitor of the protein. Besides, the average RMSD of the complexes is around 2 Å with the chlorogenic acid–DPP-IV complex revealing superior structural stability (1.75 Å) over the Diprotin A–DPP-IV complex (1.95 Å) and ellagic acid–DPP-IV complex. Interestingly, a study conducted on lac compounds against DPP-IV also showed that the best hit compounds had an average RMSD of 2 Å [42] which is consistent with the observations in the current study. Additionally, it is worth mentioning that the RMSD for the four systems reached an initial convergence at 5 ns and subsequently diverged at 45 ns. It can be noted that the RMSD of the ellagic acid complex saw an increase of around 65–70 ns, while the chlorogenic acid and Diprotin A complexes and the apoenzyme remained stable till the end of the simulation (Figure 3A).

Root mean square fluctuation has to do with the flexibility of the ligand and the attitudes of amino acid residues at the binding pocket of the enzyme. The apoenzyme in this study had an average RMSF value of 1.12 Å which is marginally lower than the 1.17 Å for the Diprotin A–DPP-IV complex and significantly higher (1.22 Å) for ellagic acid, suggesting slightly high fluctuations (Supplementary Table S1). However, the lower average RMSF value of the chlorogenic acid–DPP-IV complex (1.08 Å) compared to the unbound DPP-IV is an indication of lesser fluctuation, better flexibility, little or no distortion, and hence indicative of profound stability [43]. The RMSF plot shows similar fluctuations for all four systems (Figure 3B). However, there was a notable increase in the RMSF of the apoenzyme and the chlorogenic acid–DPP-IV complex at residues 225–250 and 475, respectively, (Figure 3B) suggesting the enhanced potential of the ligand to adapt well to the binding pockets of the proteins. This also indicates that binding of the ligand to the receptor allows better stabilisation of the fluctuation of individual amino acid residues, resulting in a more stable complex.

Unlike the RMSF, the RoG signifies the compactness of the complex formed as a result of the interaction between the ligand and enzyme [44]. In this study, both complexes of chlorogenic acid–DPP-IV and Diprotin A–DPP-IV had average RoG values of 26.95 Å and 26.96 Å, respectively, relative to 27.18 Å observed with the apoenzyme (Supplementary Table S1) and ellagic acid–DPP-IV (27.02 Å). Consistent with this observation, the apoenzyme fluctuated the most during the simulation period as evidenced by its highest value compared to the three complexes (Figure 3C and Supplementary Table S1). It was also observed that the three systems reached an initial point of convergence at 10 ns, while the apoenzyme then diverged at 25 ns (Figure 3C). The chlorogenic acid, Diprotin A and ellagic acid complexes on the other hand remained stable with similar fluctuations throughout the simulation (Figure 3C). Since high RoG values are a measure of reduced compactness of the protein–ligand complex and vice versa [45], the exhibited compactness of the three complexes (with chlorogenic acid–DPP-IV being superior) is indicative of their stability. Going by the findings of the current study on the stability, fluctuation, and compactness as expressed with RMSD, RoG, and RMSF values, it is evident that chlorogenic acid competed favorably with Diprotin A, suggesting the potential of chlorogenic acid as a probable DPP-IV inhibitor.

Solvent accessible surface area is a measure of hydrophilic and/or hydrophobicity of the enzyme’s amino acid residues on exposure to solvent or water [46], and the degree of variation of exposure could be a result of changes to the protein tertiary structure [47]. Results from the SASA analysis in this study indicated that ellagic acid had the most fluctuations during the simulation; however, consistent fluctuation patterns were noted with all four systems (Figure 3D). Similar fluctuations were observed with the chlorogenic acid–DPP-IV and Diprotin A–DPP-IV complexes, however, at a lower range than the DPP-IV. The reduced average SASA value of the chlorogenic acid–DPP-IV complex (24,324.69 Å) and Diprotin A–DPP-IV (24,625.10 Å) as established in this investigation, (Supplementary Table S1) could be suggestive of the better stability of the compounds, though chlorogenic acid had superior stability over Diprotin A. The complex with a heightened SASA value relative to the unbound protein is a consequence of its reduced solvent access for the non-polar residues preventing its stability [48] which could be attributed to the ill-effect of interruption of the hydrophobic interactions between clustered non-polar amino acid residues and the hydrophobic core during the denaturation or unfolding of the protein [47]. Analysis of the bond interactions between the chlorogenic acid, ellagic acid and Diprotin A complexes indicated that compounds and Diprotin A interacted with non-polar amino acid residues Trp622 (Figure 2A) and Trp592 (Figure 2C), respectively, thus, preventing the amino acid residue from being exposed to the aqueous surrounding as evidenced in the reduced SASA values of both complexes.

**Figure 4:**
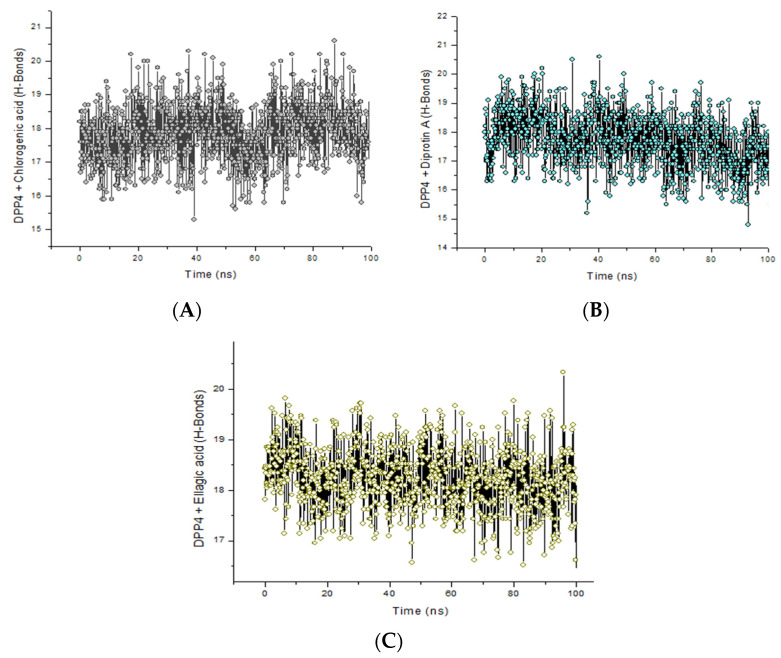
Backbone hydrogen bonds along the 100 ns simulation trajectory for DPP-IV in complex with (**A**) chlorogenic acid, (**B**) Diprotin A and (**C**) ellagic acid.

The presence of intramolecular H-bonds in a protein structure is germane in its maintenance of stability and conformation (3D) [49]. Hence, H-bond analysis in MD studies is another parameter that provides insight into the stability of the protein structures or resulting complexes formed as a result of protein–ligand binding [49,50]. H-bonds fluctuated mainly around 17–19 H-bonds in the chlorogenic acid complex (Figure 4A) and just below 17–19 H-bonds in the Diprotin A (Figure 4B) and ellagic acid (Figure 4C) complexes. All the complexes showed similar H-bond fluctuation patterns over 100 ns. However, it was noted that after 70 ns, chlorogenic acid tends to form more H-bonds with DPP-IV (in comparison to Diprotin A and ellagic acid) suggesting the reason for the higher binding energy observed with the chlorogenic acid complex, thus, enhancing the affinity of chlorogenic acid for DPP-IV. This observation agrees with the reports of Khan et al. [48] and Al-Humaydhi et al. [51] where there are consistencies in the number of H-bonds that existed between memantine–human serum albumin and galantamine–transferrin complexes post-MDS and docking findings. Above all, the H-bonding patterns of all complexes revealed that chlorogenic acid competes favorably with Diprotin A concerning the hydrogen bonding patterns over the 100 ns simulation and leads to the conclusion that chlorogenic acid could be a potential DPP-IV inhibitor.

### 3.2. Pharmacokinetics Analysis

Based on the computational analyses, it was evident that chlorogenic acid may be a suitable DPP-IV inhibitor that could compete well with a commercial inhibitor, hence was taken for further pharmacokinetics studies. Lipinski’s rule of five evaluates the druggability of a drug or molecule and it states that a compound should not possess more than five H-bond donors, 10 H-bond acceptors, a molecular weight not exceeding 500 kilodaltons and the LogP must not be more than 5. Hence, for a compound to be considered an orally available drug, it should not violate two or more of these rules [52].

The findings in the present investigation revealed that chlorogenic acid only violated one rule (having 6 OH) and Diprotin A violated none. Additionally, chlorogenic acid was found to have a low bioavailability score compared to Diprotin A, meaning that the former may be poorly absorbed into the body since the bioavailability score is a measure of the ability of the compound to pass through the systemic circulation in order to elicit its effect at the site of action [53]. The poor absorption of chlorogenic acid witnessed in this study was in contrast to an earlier study by Zhou et al. [54] where they found chlorogenic acid isolated from *Lonicerea japonicae* to be highly absorbed and eliminated following oral administration in Wistar rats. Moreover, poor absorption and excretion of chlorogenic acid have been linked to its co-administration with other components as established in the work of Qi et al. [55] where a comparative study on the bioavailability profile between chlorogenic acid isolated from *Solanum lyratum* and the extract of the same plant was studied. They found the absorption and excretion of the extract to be low following oral administration to Wistar rats, thus, attributing the reduction to the presence of other possible components in the extract. However, it is noteworthy too that a probable drug candidate is only required to have minimally 10% bioavailability [53], which is a condition already fulfilled by chlorogenic acid (11%), (Table 3) although the bioavailability score of Diprotin A was higher (55%) which is expected being a commercially available drug. Additionally, it was observed that chlorogenic acid absorption was low in the GI, which suggests that for it to be a formidable drug candidate that will pass the test of time, it might be necessary for it to be further modified to increase its GI absorption to enhance its possible function as a good DPP-IV inhibitor. Interestingly, the ability of chlorogenic acid (and Diprotin A) not to permeate the blood–brain barrier is a positive indication that it will not cause any complications, such as schizophrenia, anxiety, depression, Alzheimer’s, Parkinson’s diseases, insomnia, etc., crossing the blood–brain barrier [56].

Table 4 presents the reports of the various toxicity endpoints that may be potentiated by the test compounds. Except for the immunotoxicity metric, where chlorogenic acid was active, other tested parameters including those of Diprotin A revealed inactivity. Hence, it could be deduced that chlorogenic acid may weaken the immune system when absorbed. Similarly, the lethal dose (LD_50_) of chlorogenic acid was observed to be in excess of 5000 mg/kg while that of Diprotin A was 3000 mg/kg indicating the possibility of Diprotin A being toxic at a lower dosage, pointing to the fact that the oral administration of the latter at that concentration or above could result in the damage of target organs, such as the liver, kidney, etc.

### 3.3. In Vitro Evaluation

Diabetes mellitus is a chronic metabolic disease whose occurrence and dilapidating effects rapidly grow daily arising from chronic hyperglycaemia (elevated glucose level in the blood) as a result of insulin inaction, resistance, or both [6]. Since 764 million people are estimated would be living with the disease by 2030 [1], it then means that the negative impact of the menace would continue to increase if appropriate intervention in terms of management is not provided.

**Figure 5:**
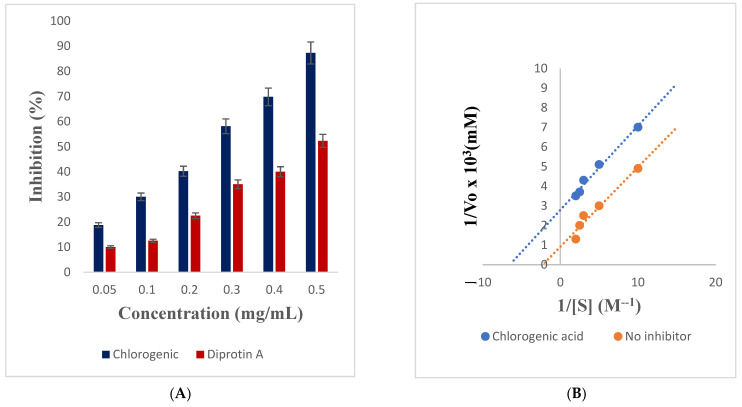
(**A**) Inhibitory effect of chlorogenic acid and Diprotin A on the activity of DPP-IV, (**B**) Uncompetitive inhibition of DPP-IV by chlorogenic acid.

Usually, OHAs and insulin (asides from the other non-convention measures, such as dietary regimen and regular exercise) are the various therapeutic interventions [5] but since they are with side effects, looking for selective, effective management and alternative therapeutic approaches revolving around GLP-1 agonists and DPP-IV inhibitors (among various other targets) is essential [6]. In the present investigation, the activity of DPP-IV was dose-dependently inhibited by chlorogenic acid and Diprotin A, with the most prominent effect observed at the highest investigated concentration in each case (Figure 5A). Judging by their IC_50_ values, chlorogenic acid had the best inhibitory activity against DPP-IV (IC_50_ value: 0.3 mg/mL) relative to 0.5 mg/mL for Diprotin A (Table 5). A further probe into the mode of inhibition of DPP-IV by chlorogenic acid revealed that the enzyme was uncompetitively inhibited (Figure 5B), with the V_max_, K_m_, and K_cat_ values decreasing from 1.09 × 10^−3^ M/min, 4.42 × 10^−4^, and 6.29 × 10^−4^ M/min, to 3.59 × 10^−4^ M/min, 1.54 × 10^−4^ M and 2.07 × 10^−4^ M/min, respectively, in the presence of chlorogenic acid (Table 5). The implication of this is that chlorogenic acid binds to a site not far from the active site of the enzyme. Incretin (hormone) dually functions by allowing the body to secrete insulin when required while at the same time mopping up excess glucose from the systemic circulation when not required. However, the action of this hormone is terminated by the DPP-IV enzyme, hence, DPP-IV inhibitors, such as Diprotin A block the activity of the DPP-IV enzyme that destroys this vital hormone. The inability of chlorogenic acid to compete with the substrate at binding at the active site of the enzyme as reflected in the uncompetitive inhibition could suggest its possible antidiabetic effect since binding to the enzyme–substrate complex (ESC) might still hinder the activity of the enzyme. Uncompetitive inhibitors bind only to the ESC and not the unbound enzyme [57]. The inhibitor-bound ESC forms when there is a high concentration of substrate present in the environment preventing the release of the product when bound resulting in decreased V_max_. The inhibitor-bound complex also results in the decrease of ESC concentration which creates a shift towards the formation of the additional ESC to equilibrate the system. The shift results in fewer free enzymes available and more enzymes in the forms of enzyme–substrate and enzyme–substrate–inhibitor complexes [57]. Theoretically, a decrease in the free enzyme corresponds to an enzyme with a greater affinity for the substrate. Thus, uncompetitive inhibition results in both decreased V_max_ and K_m_. Since the formation of product is hindered, this also leads to decreased product turnover, and therefore, a decreased K_cat_ value. Some studies established different kinetics of inhibitions (competitive and non-competitive) of DPP-IV by phenolic compounds, such as luteolin, apigenin, resveratrol, flavone, and a phenethylphenylphthalimide analogue, etc., obtained from citrus, berry, etc. [13,58]. However, a peptide (WLQL) among other novel peptides isolated from *Pelodiscus sinensis* was found to have an uncompetitive inhibition against DPP-IV [59] as observed in the study.

## 4. Conclusions

Computational studies are invaluable and significant in screening a library of compounds with potential therapeutic effects in drug discovery and development as evidently shown in this study. Judging by the findings from the computational analyses in this study, chlorogenic acid established superior interaction and affinity with DPP-IV above Diprotin A. Additionally, in vitro investigation revealed the uncompetitive inhibition of DPP-IV by chlorogenic acid indicating both the in-silico result and in vitro assessment being in tandem with one another, demonstrating the potential of chlorogenic acid as a viable candidate in the management of T2D via the inhibition of DPP-IV. Since medicinal plants and/or natural product compounds have continued to find their place as probable alternatives in disease control and management, the laudable activity exhibited by chlorogenic acid in this study attests to the submission about their superiority in the therapeutic effectiveness of natural compounds over some conventional drugs. It is evident that with these findings, diabetes researchers or stakeholders, and sufferers of T2D can be hopeful for the development of chlorogenic acid as a suitable alternative for diabetes management. Further studies are suggested on the optimisation of the compound to enhance its druggability in diabetes therapy as well as in vivo evaluation of its antidiabetic property.

## Figures and Tables

**Table 1:** Binding energy scores of phenolic acids and dipeptidyl peptidase-IV formation.

Compounds	Score (kcal/mol)
Chlorogenic acid	−9.0
Diprotin A	−6.6
Ellagic acid	−7.8
Gallic acid	−6.4
Caffeic acid	−6.3
O-coumaric acid	−6.3
Olivetolic acid	−6.3
Umbellic acid	−6.3
Isoferulic acid	−6.2
M-coumaric acid	−6.2
P-coumaric acid	−6.1
Protocatechuic acid	−6.1
Orsellinic acid	−6.1
Ferulic acid	−6.0
Sinapic acid	−6.0
Hypogallic acid	−6.0
Vanillic acid	−5.9
Beta-resorcinolic acid	−5.7
Salicyclic acid	−5.8
Syringic acid	−5.7
Veratric acid	−5.7
Gentisic acid	−5.6
Benzoic acid	−5.4

**Table 2:** Energy components analyses of the molecular dynamics simulation of the compounds.

Energy Components (kcal/mol)					
Complexes	ΔE_vdW_	ΔE_elec_	ΔG_gas_	ΔG_solv_	ΔG_bind_
DPP-IV + Chlorogenic acid	−28.64 ± 6.84	−49.38 ± 21.85	−78.03 ± 19.96	52.29 ± 15.63	−25.74 ± 6.45 ^a^
DPP-IV + ellagic acid	−25.33 ± 4.74	−58.51 ± 18.28	−83.84 ± 16.51	69.30 ± 11.19	−24.54 ± 6.31 ^a^
DPP-IV + Diprotin A	−20.32 ± 3.43	−261.44 ± 40.36	−281.76 ± 40.63	269.32 ± 36.75	−12.44 ± 5.48 ^b^

DPP-IV: dipeptidyl peptidase-IV; ΔEvdW: van der Waals energy; ΔEelec: electrostatic energy; ΔEgas: gas-phase free energy; ΔGsolv solvation free energy; ΔGbind: total binding free energy. Values with different superscript letters along the same row are significantly (*p* < 0.05) different from each other.

**Table 3:** Drug-likeness and absorption, distribution, metabolism, and excretion (ADME) properties of chlorogenic acid and Diprotin A based on gastrointestinal absorption, blood–brain barrier permeation, possible P-gp substrate, and possible cytochromes enzyme inhibition.

Property	Chlorogenic Acid	Diprotin A
Lipinski’s rule of five	Yes; 1 violation: NH or OH > 5	Yes; 0 violations
Bioavailability score	0.11	0.55
GI absorption	Low	High
BBB permeant	No	No
P-gp substrate	No	Yes
CYP1A2 inhibitor	No	No
CYP2C19 inhibitor	No	No
CYP2C9 inhibitor	No	No
CYP2D6 inhibitor	No	No
CYP3A4 inhibitor	No	No

CYP: Cytochrome.

**Table 4:** Toxicity classifications and LD_50_ of chlorogenic acid and Diprotin A.

Classification	Chlorogenic Acid	Diprotin A
Hepatotoxicity	Inactive	Inactive
Carcinogenicity	Inactive	Inactive
Immunotoxicity	Active	Inactive
Mutagenicity	Inactive	Inactive
Cytotoxicity	Inactive	Inactive
LD_50_ (mg/kg)	5000	3000

LD: Lethal dose.

**Table 5:** Inhibitory effect (IC_50_) and enzyme kinetic parameters of chlorogenic acid against DPP-IV.

Inhibitor	IC_50_ (mg/mL)	V_max_ (M/min)	K_m_ (M)	K_cat_ (M/min)
Chlorogenic acid	0.3 ± 0.02 ^a^	3.59 × 10^−4 a^	1.54 × 10^−4 a^	2.07 × 10^−4 a^
Diprotin A	0.5 ± 0.02 ^b^	ND	ND	ND
No inhibitor	ND	1.09 × 10^−3 b^	4.42 × 10^−4 b^	6.29 × 10^−4 b^

ND: Not determined. Values (n = 3) with different superscript letters along the same row are significantly (*p* < 0.05) different from each other.

## Data Availability

The data presented in this study are available in the article.

## Supplementary Materials

The following supporting information can be downloaded at: https://www.mdpi.com/article/10.3390/metabo12100937/s1, Table S1: Average RMSD, RMSF, RoG and SASA values of DPP-IV in complex with chlorogenic acid and Diprotin A.
