# Supplementary material for: Cheminformatics Identification and Validation of Dipeptidyl Peptidase-IV Modulators from Shikimate Pathway-Derived Phenolic Acids towards Interventive Type-2 Diabetes Therapy
Source: Metabolites. 2022 Oct 2;12(10):937. doi: 10.3390/metabo12100937 (PMC9608993; doi:10.3390/metabo12100937)
Supplement: Supplementary file 1 [file metabolites-12-00937-s001.zip › metabolites-1953554-supplementary.pdf]

Table S1. Average RMSD, RMSF, RoG and SASA values of DPP-IV in complex with chlorogenic acid and Diprotin A.

Complexes	Average RMSD (Å)	Average RMSF (Å)	Average ROG (Å)	Average SASA (Å)
**DPP-IV + Chlorogenic acid**	1.76 ± 0.14	1.08 ± 0.50	26.95 ± 0.08	24324.70 ± 328.06
**DPP-IV + Diprotin A**	1.96 ± 0.20	1.18 ± 0.56	26.96 ± 0.10	24636.10 ± 371.14
**DPP-IV**	1.69 ± 0.16	1.13 ± 0.78	27.18 ± 0.10	24982.98 ± 367.09
